# Sustainable Production of Biodiesel Using Waste Jatropha curcas Shells as a Heterogeneous Catalyst

**DOI:** 10.1021/acsomega.4c06782

**Published:** 2025-06-09

**Authors:** Indira Tobío-Pérez, Magín Lapuerta, Laureano Canoira, Samuel Lalthazuala Rokhum, Ramón Piloto-Rodríguez

**Affiliations:** 1 Department of Energy & Fuels -Tecminergy, Escuela Técnica Superior de Ingenieros de Minas y Energía, 16771 Universidad Politécnica de Madrid, Ríos Rosas 21, Madrid 28003, Spain; 2 Escuela Técnica Superior de Ingeniería Industrial, 16733 Universidad de Castilla La-Mancha, Avda. Camilo José Cela s/n, Ciudad Real 13071, Spain; 3 Department of Chemistry, 28687 National Institute of Technology Silchar, Silchar 788010, India

## Abstract

In this study, the feasibility of using a calcined Jatropha curcas shell (JCS) as a solid catalyst for biodiesel production was investigated. Thermogravimetric analysis (TGA) was applied to the precursor biomass to evaluate the stages of the thermal decomposition processes and to identify proper temperature intervals for biomass calcination, aiming to decarbonize to form oxides that have proven to behave as effective active sites. The kinetic parameters were defined by applying Kissinger’s isoconversional method. The activation of the precursor biomass was carried out at three calcination temperatures (700, 800, and 900 °C) and two calcination times (2 and 4 h) in order to evaluate the influence of these operating conditions on the structure, morphology, and catalytic activity of the catalyst. The catalyst was characterized by the Brunauer–Emmett–Teller method (BET), scanning electron microscopy with energy-dispersive X-ray (SEM/EDX), and X-ray diffraction (XRD). The effectiveness of the catalyst was assessed by oil’s transesterification at 60 °C, 2 h of reaction, and a 15:1 alcohol/oil molar ratio. The effects of catalyst loading (1, 3, and 5 wt %) and activation temperature of the catalyst on the yield and conversion to fatty acid methyl esters (FAMEs) were studied. The highest values of conversion and FAME yield were obtained with a 3% catalyst load (calcined at 800 °C for 2 h). Ester content and fatty acid profile were obtained by chromatographic analysis. The results of this study demonstrate that calcined JCS has a high potential as a heterogeneous catalyst for biodiesel production.

## Graphical abstract

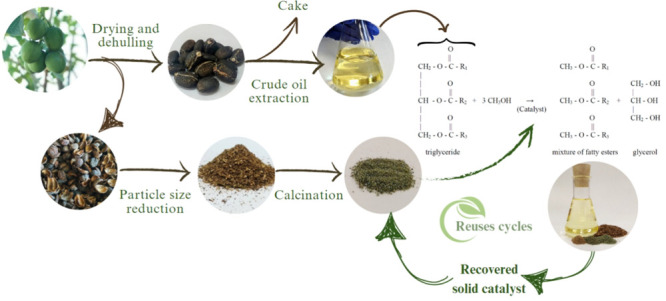

## 1 Highlights

Calcined *Jatropha* shell has strong potential as a catalyst for biodiesel production.

The catalyst surface shows an irregular morphology with changes in the porosity.

According to the EDX analysis, the main elements present are K, Ca, and O.

XRD patterns show the presence of oxides of metals, such as K, Ca, Mg, and Si.

The best values for oil conversion, FAME yield, and ester content were obtained using a 3% catalyst loading calcined at 800 °C for 2 h.

## 2 Introduction

The production and excessive use of fossil fuels are major concerns for modern societies as they lead to unprecedented global warming and environmental pollution. The most viable alternative is the production of environmentally friendly renewable fuels, among which biodiesel, a marketable and well-known product worldwide, represents the main option for partial or total diesel fuel substitution.^1,2^ Currently, biodiesel is seen as a much safer fuel with less negative environmental impact compared to conventional fuels. It is also a renewable fuel, and its availability and potential reduction of production costs are some of its associated advantages. It is mainly produced from vegetable oils such as palm, soybean, rapeseed, coconut oil, J. curcas and Moringa oleifera among others,^3–5^ but it can also be produced from waste cooking oil^6,7^ or industrial byproducts.^8,9^

A catalyst is essential for biodiesel production. In this respect, there are two general types of catalysts, conventional homogeneous catalysts and heterogeneous ones. Homogeneous basic catalysis is the most used in research and industry for biodiesel production, with sodium and potassium hydroxides being the most widely used catalysts. Homogeneous catalysts have a variety of applications in the biodiesel industry due to their high catalytic activity and availability. Homogeneous base catalysts have been extensively investigated and implemented in the industry but are strongly affected by the presence of free fatty acids (FFAs). FFAs interfere with the transesterification reaction by forming surfactant molecules, which promote the formation of stable emulsions during the purification stage. Pretreating the oil through acid esterification is a common strategy to reduce the FFA content. The use of homogeneous catalysts in the production of biodiesel has some drawbacks, such as difficult catalyst recovery, low quality of glycerin obtained as a byproduct, and corrosion effects.

For these reasons, the development of heterogeneous catalysts, also known as solid catalysts, to produce biodiesel is very important. A heterogeneous catalyst refers to a form of catalyst whose phase differs from the reactants of the chemical reaction^4^. The reaction mechanisms in heterogeneous catalysis are based on the adsorption of triglycerides (TGs) on the catalyst surface by the interaction of double-bonded oxygen, forming a carbonate. In the case of metal oxide-based catalysts, the active sites, primarily basic sites generated by alkaline earth metal oxides (CaO, MgO, and K_2_O), play a crucial role in facilitating the reaction. These oxides provide strong Lewis basic sites that promote the deprotonation of methanol, generating the reactive methoxide species necessary for the transesterification reaction. The authors described these mechanisms in a previous comprehensive review.^10^

Compared to homogeneous catalysts, heterogeneous catalysts are less corrosive, and the products are obtained with higher purity; they can be easily recovered and reused, simplifying the process thanks to the reduction of washing steps. All these factors contribute to reduce the biofuel’s production cost. Nevertheless, the use of solid catalysts has several drawbacks, such as lower catalytic activity, the catalytic site leaching, and, in certain cases and work conditions, the product can be easily contaminated.

The use of biomass residues or biodiesel production byproducts as precursors to produce heterogeneous catalysts not only reduces the cost of the catalyst but also contributes to the reuse of the biomass residues generated by different human and agricultural activities.^10^ Biomass catalysts are biodegradable, have lower toxicity, include several mineral components, and are adjustable in pore size and chemical structure. J. curcas is a known and well-reported feedstock for biodiesel production, mainly at the local scale. In developing countries, this plant is one of the main nonedible feedstocks for transesterification.^11,12^ Many Jatropha strains produce a nonedible oil due to its content of phorbol ester, limiting also the use of the transesterification byproducts for certain uses.

The production of biodiesel from Jatropha generates a significant amount of seed oil cake and shell. The seed cake can be used either as a biofertilizer^13^ or as feedstock for biogas and charcoal production (see Figure 1). However, Jatropha shell, which represents barely 40% of the fruits,^12^ has more restricted uses in the biodiesel industry. Even when the production of biodiesel from J. curcas oil is focused on local-scale production, local facilities need, among other requirements, the use of a homogeneous basis catalyst, typically NaOH or KOH, which is not recovered during the chemical process. Besides this main drawback, it must be purchased in the market, representing a high constrain in the frame of a third world regulated economy.

**Figure 1:**
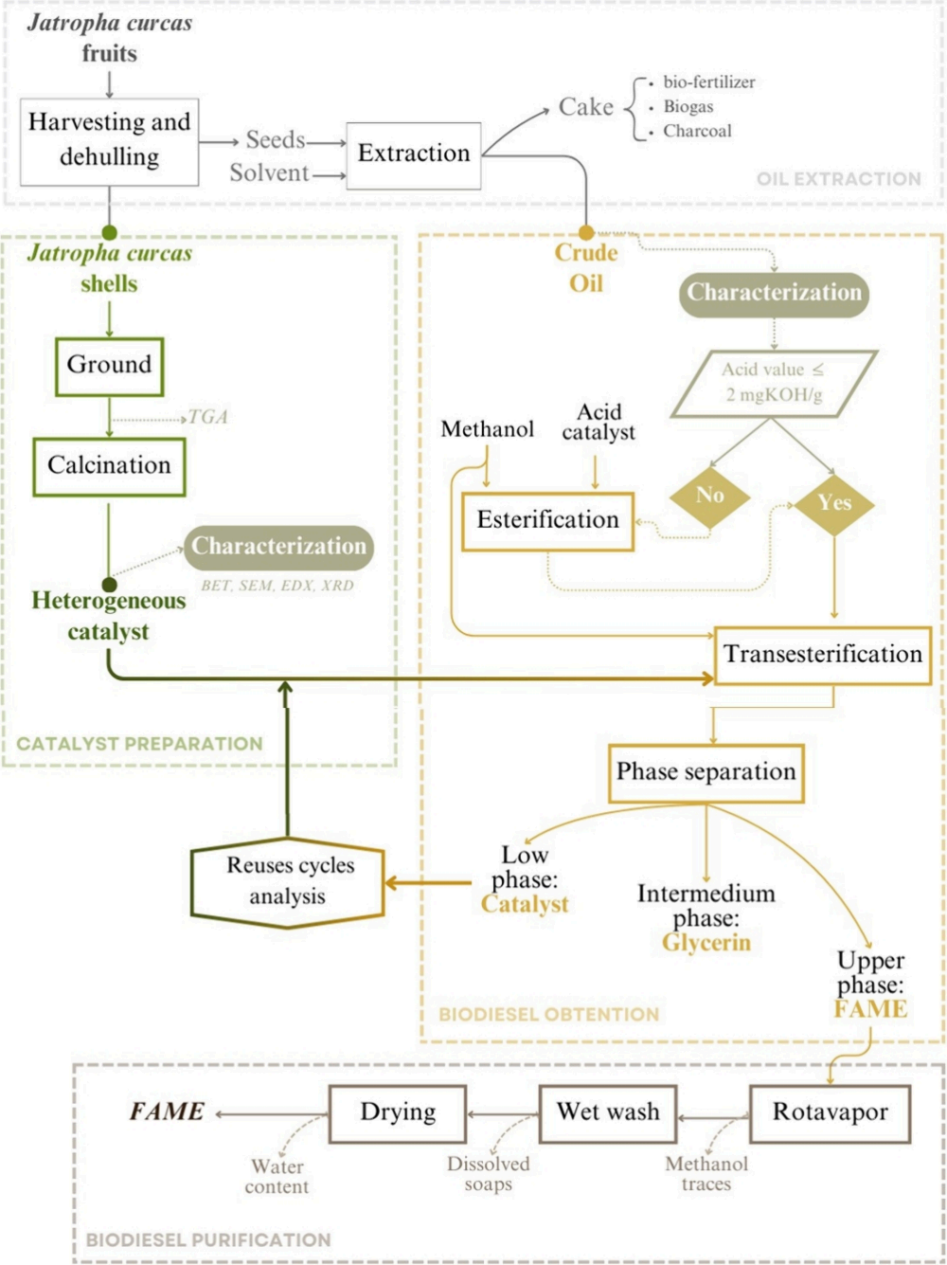
Diagram of the biodiesel production process.

The production of a solid catalyst based on a biomass precursor, which is a byproduct of the local biodiesel industry, is a key factor to reduce biofuel production cost and to contribute to a circular economy, with reduction of the environmental impact associated with the use of chemicals, both during the upstream and downstream processes. The aim of this work is to develop a solid catalyst based on the use of a J. curcas shell (JCS) as a biomass precursor, which is activated by calcination. To assess the catalyst efficiency for transesterification reaction, different experiments have been conducted at different operating conditions and several analytical techniques have been applied, with and without thermal activation, such as TGA, BET method, SEM, EDX, and XRD. Finally, preliminary assessment of catalyst effectiveness is analyzed by gas chromatography (GC-FID) applied to the transesterification product. Figure 1 shows a diagram of the experimental process. The oil extraction stage is not the subject of this study; however, it is important to include them in the diagram as they illustrate the circular economy approach intended to be achieved with the research.

## 3 Materials and Methods

### 3.1 Materials and Catalyst Preparation

Jatropha shell biomass was collected from a J. curcas biodiesel production facility and was ground and reduced to less than 1.5 mm particle size. Figure 2 shows the appearance of the biomass under study before and after the size reduction.

**Figure 2:**
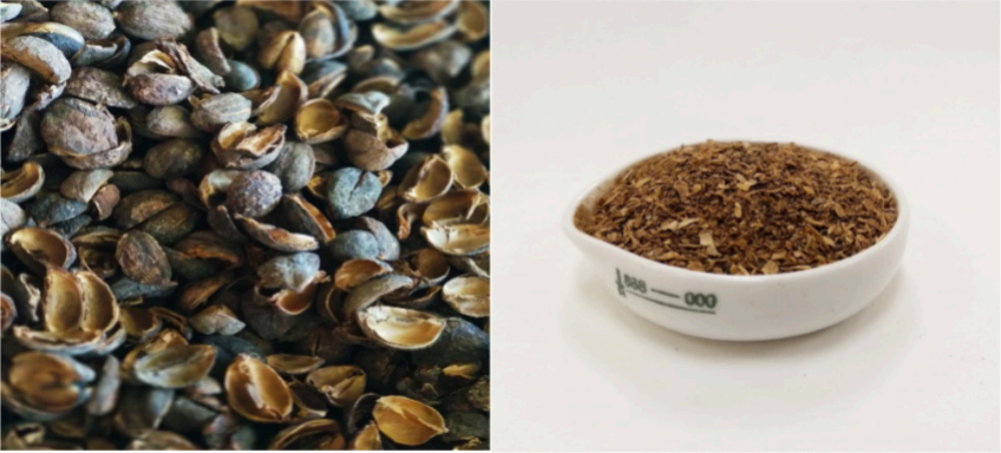
J. curcas shell before and after particle size reduction (≤1.5 mm).

TGA was applied to the noncalcined biomass (JC-0) to identify and assess the stages of thermal decomposition processes. A TGA Q500 V6.7 Build 203 thermogravimetric balance was used. The technique was performed in air and inert (N_2_) atmosphere (90 mL/min) at three heating rates: 5, 10, and 20 °C/min up to 1000 °C. The application of TGA led to identifying suitable temperatures and temperature intervals for JCS biomass calcination, as well as to defining the activation energy related to the thermal decomposition process.

Concerning the estimation of the apparent activation energy, the TGA data were processed according to the isoconversional method, which does not require previous knowledge of the reaction model.^14^ Thermal decomposition data were gathered at three different heating rates (5, 10, and 20 °C/min) under the above-described thermogravimetric experimental conditions. Thermal data fitting was conducted at a linear heating rate, according to the isoconversional method. The kinetic method selected for fitting and determination of apparent activation energy at different conversion degrees was the Kissinger’s method, using eq 1. The characteristics of this method are detailed by Cai et al.:^15^

$$\ln\left(\frac{\beta_{i}}{T_{\alpha ,i}^{2}}\right)=\ln\left(\frac{R\cdot A_{\alpha }}{E_{\alpha }}\right)+\ln\left[n{\left(1-\alpha \right)}^{n-1}\right]-\frac{E_{\alpha }}{R\cdot T_{\alpha ,i}}$$ 1

where *E*_α_ is the apparent activation energy, *n* the reaction order, *A*_α_ the apparent Arrhenius pre-exponential factor, β the heating rate, *T* the temperature, *R* the universal gas constant, and α the reaction conversion degree. The conversion degree was determined from eq 2:

$$\alpha =\frac{m_{o}-m_{t}}{m_{o}-m_{f}}$$ 2

where *m*_*o*_ is the initial biomass mass, *m*_*t*_ is the biomass mass at time *t*, and *m*_*f*_ is the final biomass mass after the combustion stage.

The activation of the precursor biomass was carried out through calcination in an oxygen atmosphere, which is the most widely used method for obtaining biomass-based catalysts. Calcination was carried out in a Carbolite AAF 1100 muffle furnace with a Carbolite type 3216 P1 temperature controller, at different operating conditions, to assess their influence on the characterization and efficiency of the catalyst. Samples of Jatropha calcined shell at three different calcination temperatures (700, 800, and 900 °C) and two calcination times (2 and 4 h) were prepared.

The calcination temperatures were selected according to the results of TGA. Figure 3 shows the aspect of the calcined samples (no visual differences were found by comparing the process at different calcination times). Afterward, the calcined samples were placed in an oven and kept at 80 °C overnight. Once the oven drying stage was complete, the samples were cooled to room temperature in a desiccator, preventing it from absorbing moisture or from interacting with CO_2_.

**Figure 3:**
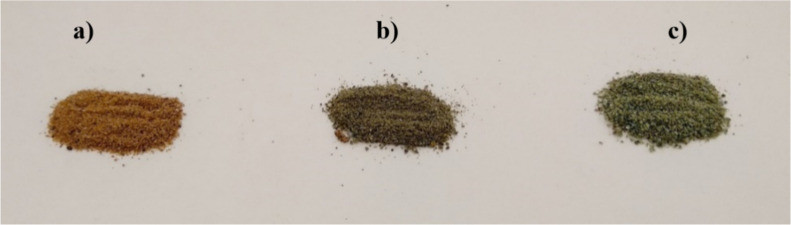
J. curcas shell samples calcined for 2 h at 700 (a), 800 (b), and 900 °C (c).

For the base biomass precursor and calcined potential Jatropha shell solid catalysts, seven different samples were prepared, as reported in Table 1.

**Table 1:** Calcination Conditions and Nomenclature of the Catalyst Samples Selected for This Study

sample	calcination temperature	calcination time	nomenclature
1	no calcination		JC-0
2	700	2	JC-700-2
3	800	2	JC-800-2
4	900	2	JC-900-2
5	700	4	JC-700-4
6	800	4	JC-800-4
7	900	4	JC-900-4

### 3.2 Catalyst Characterization

Characterization techniques such as SEM with X-ray dispersive analysis (SEM-EDX) were applied to the samples to analyze the surface morphology and to identify the elements presented in the material and their concentration. X-ray diffractometry (XRD) was developed to obtain information about the crystallinity of the samples. The specific surface area, diameter, and pore volume were determined by applying the Brunauer–Emmett–Teller (BET) method to the samples. The equipment was a Micromeritics instrument, using nitrogen as the adsorptive gas, with ambient saturation pressure. Barely 0.06 g of biomass sample and three replicates per biomass treatment were tested. Raw JC-0 and calcined samples at 700, 800, and 900 °C were analyzed.

The morphological characteristics of raw and calcined JCS were examined by a Scanning Electron Microscope GeminiSEM 500 equipped with an EDX spectrometer and X-max detector (Oxford-instruments, INCA 400, UK), used for the determination of the elemental composition of the biomass precursor and calcined samples at different calcination temperatures and time.

The samples were placed on the SEM stub using double-sided carbon tape, inserted into the chamber, 2 kV accelerating voltage, 5.9 mm working distance, scan speed 7, and ×1000 magnification.

The XRD study was performed on a Panalytical X’Pert MPD diffractometer equipped with a LynxEye XE-T position sensitive detector with CuKα_1_ radiation (1.5418 Å) over a 2θ range from 10° to 90° with a step size of 0.02°.

### 3.3 Synthesis and Characterization of Biodiesel

To evaluate the effectiveness of the heterogeneous catalyst, the transesterification of unrefined J. curcas oil (JCO) was compared to that of high-oleic-sunflower-refined oil (SFO). The physicochemical properties of both oils are summarized in Table 2. In the specific case of JCO, a previous esterification reaction was conducted before transesterification, due to the high acid value exhibited by this oil.

**Table 2:** Physicochemical Properties and Fatty Acid Profile of Oils^a^

**physicochemical properties**	**SFO**	**JCO**
acid value (mgKOH/g)	0.28	7.76
water content (wt %)	0.03	0.06
kinematic viscosity at 40 °C (mm^2^/s)	37.62	34.26
density at 15 °C (g/cm^3^)	0.910	0.899
fatty acid profile, w/w % (Cx:y)*		
palmitic (C16:0)	4.444	13.745
palmitoleic (C16:1)		0.693
stearic (C18:0)	3.275	6.477
oleic (C18:1)	81.608	43.402
linoleic (C18:2)	5.67	29.706
linolenic (C18:3)		0.234
arachidic (C20:0)		1.776
behenic (C22:0)	1.086	3.249
lignoceric (C24:0)	3.917	0.718
saturated	12.722	25.965
unsaturated	87.278	74.035
molar mass (g/mol)	890.90	879.24

a SFO = sunflower refined oil; JCO = unrefined J. curcas oil; (C*x*:*y*) = “*x*” is the number of carbon atoms “*C*” in the molecule, and “*y*” is the number of unsaturations.

#### 3.3.1 First Reaction Stage

In the first reaction stage for JCO, two esterification methods using different acids were applied to select the one that resulted in a lower acid value. The first method consisted of the esterification of JCO with *p*-toluenesulfonic acid (oil–acid mass ratio, 200:1) solved in methanol (molar ratio of methanol–oil, 6:1) following the procedure proposed in Canoira et al.^16^ The reaction mixture was kept at 60 °C with stirring at 600 rpm for 4 h. At the end of the reaction, calcium oxide (mass ratio oil–calcium oxide, 50:1) was added to the reactor to neutralize the acid catalyst and to eliminate the water produced in the esterification, forming insoluble calcium hydroxide. The reaction mixture was cooled to room temperature and filtrated to eliminate the calcium salts. The resulting mixture of JCO and methanol was used for the next transesterification step without any further treatment.

The second esterification method was carried out with sulfuric acid (96% purity) at a concentration of 1% (w/w), with a methanol molar ratio of 8:1, at 55 °C for 90 min. After the reaction time, the mixture was allowed to settle in a separatory funnel for 24 h to observe the separation of two phases. The upper phase (aqueous phase with methanol and the remaining catalyst) was separated from the lower phase (esterified oil) to react the latter through the second stage of transesterification.

The FFA conversion values (*C*_FFA_) for the esterification reactions were obtained by comparing the acid values before and after the reaction. eq 3 was used, where *A*_*i*_ is the initial acid value of the oil and *A*_*e*_ corresponds to the acid value of the esterified oil:

$$C_{\mathrm{FFA}}=\frac{A_{i}-A_{e}}{A_{i}}\times 100$$ 3

#### 3.3.2 Second Reaction Stage

In the transesterification process, the SFO or JCO esterified was heated at 60 °C for 2 h with a 15:1 methanol/oil molar ratio, in a 250 mL round-bottom flask using a temperature-controlled magnetic stirrer. The effects of catalyst loading (1, 3, and 5 wt %) and activation temperature of catalyst (700, 800, and 900 °C) on the yield and conversion to FAME were studied. The FAME yield (η_FAME_) and oil conversion (*C*_oil_) values were determined with eqs 4 and 5, respectively:

$$\eta_{\mathrm{FAME}}=\frac{\frac{m_{b}\cdot f_{\mathrm{FAME}}}{{\mathrm{MM}}_{\mathrm{FAME}}}}{3\cdot \frac{m_{\mathrm{oil}}}{{\mathrm{MM}}_{\mathrm{oil}}}}\times 100$$ 4

$$C_{\mathrm{oil}}=\frac{m_{\mathrm{oil}}-\left[m_{b}\cdot \left(1-f_{\mathrm{FAME}}\right)\right]}{m_{\mathrm{oil}}}\times 100$$ 5

where *m*_oil_ is the oil mass to be transesterified (g); *m*_*b*_ is the biodiesel mass (g); *f*_FAME_ is the mass fraction of FAME determined using EN 14103; η_FAME_ is the FAME yield (%); *C*_oil_ is the oil conversion (%); and MM_oil_ is the molar mass of the oil (see Table 2) and MM_FAME_ is the molar mass of FAME.

The obtained product underwent a purification procedure designed to remove moisture, methanol traces, soaps, and other impurities. First, biodiesel was transferred to a rotary evaporator to eliminate the excess methanol. Then, a conventional washing method was employed, involving the separation of soaps from the fuel using preheated water at 50 °C. Following stirring, soap formation was observed, and the mixture was allowed to settle for approximately 1 h. The purified biodiesel remained at the top, while the water containing dissolved soaps settled at the bottom of the funnel. This washing process was iterated until the separated water became clear and attained a neutral pH. Subsequently, the biodiesel proceeded to a drying stage to eliminate the residual water content. This involved a gradual heating process to facilitate the evaporation of water present in the fuel.

The equipment was a Gas Chromatograph HP 5890 Series II. The column was a HP-Wax (30 m × 0.32 mm × 0.15 μm) with a flow rate of 2 mL/min; split 60:1, 0.45 min; and injection volume of 1 μL. A Flame Ionization Detector set at 250 °C was used, and temperature programming was 60 °C (2 min), 10 °C/min to 200 °C, and 5 °C/min to 240 °C (7 min). The total ester content and ester profile were determined using EN 14103.

## 4 Results and Discussion

### 4.1 Catalyst Preparation

The TGA results indicate that the JCS sample is thermally degraded in three main stages, as shown in Figure 4. The TG and DTG curves represent weight loss and weight loss rates, respectively. In the first stage, moisture removal occurs at temperatures below 150 °C. Consequently, a weight loss of about 10% occurs. The maximum rate of weight loss (40–50%) occurs during devolatilization at a temperature range between 200 and 380 °C. The decomposition of hemicellulose and cellulose has been associated with this stage in the literature.^17,18^ The fixed carbon decomposition begins at temperatures above 400 °C, while heavier volatiles decompose. The lignin decomposition has been identified generally with the third stage, which occurs gradually over a wide temperature range.^19^ The characteristic peak of this combustion stage can be seen in experiments carried out under an oxidizing atmosphere only. The joint analysis of the thermograms in an inert and oxidizing atmosphere allows estimation of the beginning of the third stage. The presence of small peaks between 580 and 600 °C could be due to an incomplete combustion of the biomass between 400 and 450 °C. A similar behavior to that shown in Figure 4 was observed at other heating rates.

**Figure 4:**
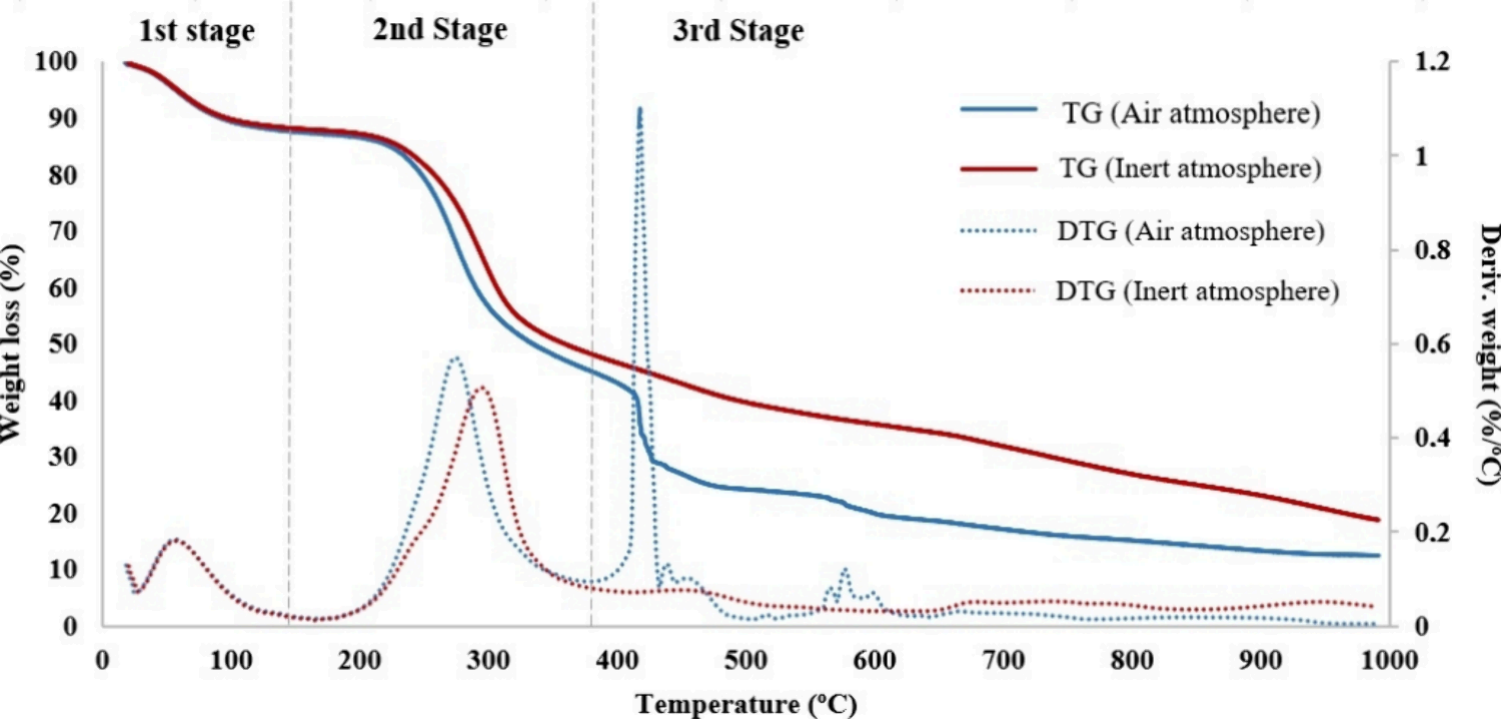
TG and DTG of the JCS at 5 °C/min in inert and air atmosphere.

The TG and DTG curves agree with those reported by Odetoye et al.^17^ These authors also tested Jatropha fruit shells, but unlike the current study, the TGA only recorded up to 600 °C. However, the authors did not report the appearance of a smaller fourth peak, around 580 °C. Nonetheless, the presence of small peaks after the three characteristic peaks of lignocellulosic biomass was reported by Romulia et al.^18^

If the thermograms published in the literature that used JCS are compared, it turns out that the TG and DTG curves obtained do not necessarily match in terms of the number of peaks observed, the temperature and intensity of the peaks, and the weight loss rate.^17,18,20,21^ Different factors can influence the form of thermograms, from the origin and biomass composition, even when it is the same species and genus, to the operating conditions. Zhang et al.^22^ showed that higher hemicellulose content leads to a lower devolatilization onset temperature, which causes a shift of the peak toward lower temperatures. Also, a higher amount of cellulose and lignin in the sample means that a higher temperature is needed to decompose. Higher ash and fixed carbon contents in the biomass lead to a higher residual mass, thereby lowering the total weight loss in the combustion process.

Figure 5 shows the behavior of the TG and DTG curves in an oxidizing atmosphere at the three heating rates under study. The weight loss profile (Figure 5a) shows a higher slope in the section of the curve corresponding to the devolatilization stage, associated with higher reaction rates when the heating rate increases.^20^ Consequently, the intensity of the peaks in the DTG curves increases as the heating rate increases (Figure 5b). In the DTG curves, a shift toward higher temperature of the characteristic peaks of the lignocellulosic biomass can be observed with increasing heating rate. This trend was also reported by Sricharoenchaikula et al.^20^ This can be explained because at a high heating rate, heat may not be able to be transferred effectively to the inner layer of the raw material, resulting in a higher temperature gradient between the inner and outer layers of the biomass particles.

**Figure 5:**
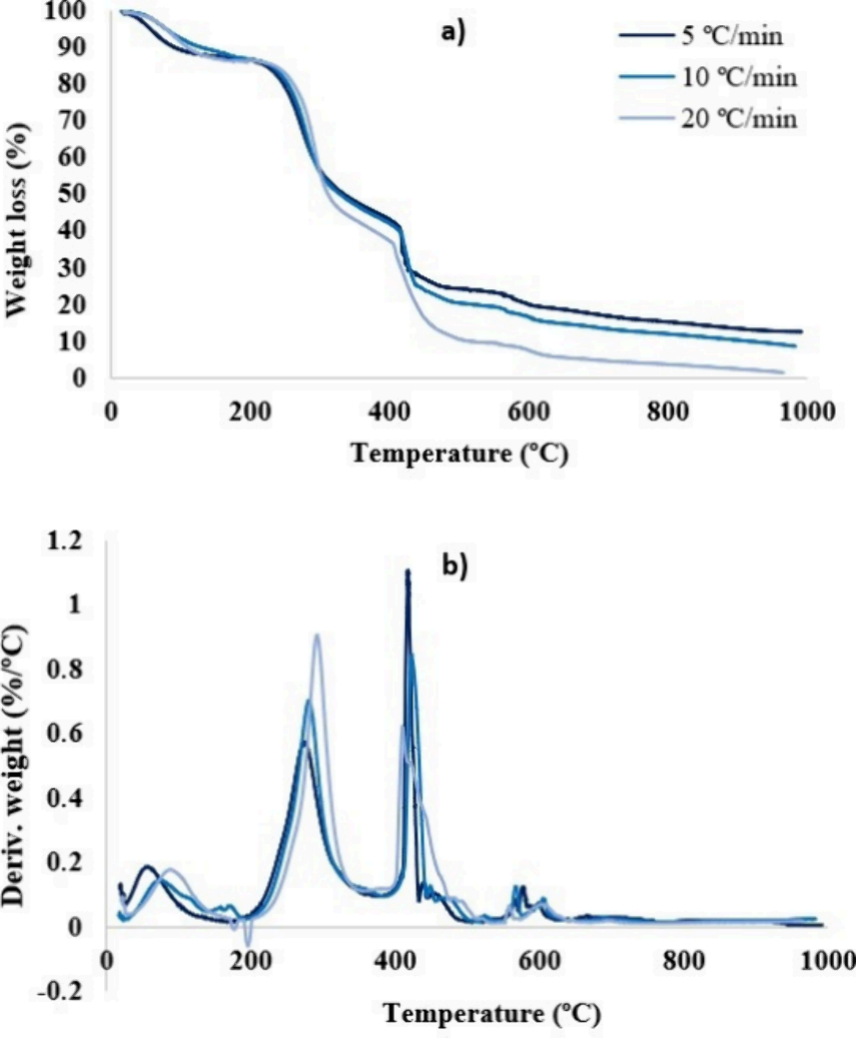
TG (a) and derivate TG (DTG) (b) in an oxidizing atmosphere

The decomposition of carbonates into oxides requires more energy than that released by combustion. It usually occurs after the fixed carbon combustion stage and is evidenced as a loss of weight in both inert and oxidizing atmospheres. The presence of oxides (CaO, K_2_O, MgO, SrO, among others) in biomass enhances the catalytic activity.^10,23,24^ As the interest lies in using the JCS as a precursor of a solid catalyst, the temperature used to activate the biomass by calcination must be higher than the temperature at which the last peak of the DTG curve appears. It is assumed that after this event, the formation of oxides gradually begins, favoring the appearance of active sites on the catalyst surface.

The results obtained concerning the estimation of the apparent activation energy as a function of the conversion degree (α) are listed in Figure 6. According to it, there are two conversion ranges where the kinetics leads to negative activation energy, with minimum values at 0.7 and 0.85. For these α values, the average temperature is around 417 °C, and the other is beyond 460 °C. These could be associated with the fact that, at those temperatures, an exothermic K_2_O/CaO structural transformation takes place, leading to negative activation energy due to significant changes in the reaction rate.^25^ Negative activation energy should be associated with spontaneous reactions, but in this case, an intermediate regime can lead to a change in the Arrhenius slope. Beyond this regime (from 0.65 conversion onward), the weight loss rate decreases as the volatile components are depleted, previously to the decomposition of components that require higher temperatures.^26^

**Figure 6:**
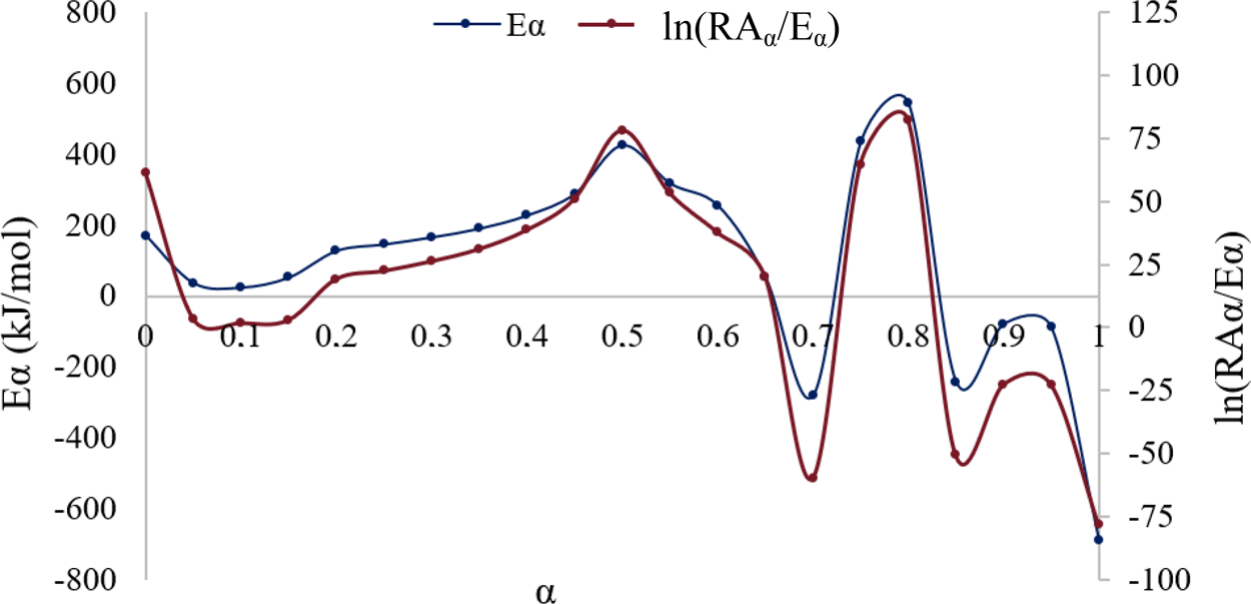
Eα and ln (RAα/Eα) as a function of conversion degree for JC shell calcination.

The expected behavior of the apparent activation energy with the biomass conversion should be an overall increase, although with slight decrease intervals.^27^ Such an increase is clearly observed until half of the mass conversion in this research. After that, the fluctuation in the activation energy values could be governed by previously explained phenomena. Fluctuations of apparent activation energy leading to negative values have been widely reported over 0.4–0.6 conversion degree for different types of biomass.^26,28,29^

### 4.2 Catalyst Characterization

The results of the BET technique application are shown in Table 3. According to the results, the activation process shows increases in both surface area and pore size. Specifically, as the calcination temperature rises in the range of 700 to 800 °C at a calcination time of 2 h, a significant increase in surface area and pore width is observed. However, a decrease in these parameters is noted upon reaching a temperature of 900 °C. Nevertheless, extending the calcination time to both 700 and 800 °C results in a noteworthy reduction in surface area and pore width. Despite that increase in calcination time at 900 °C leading to an increase in surface area, it remains lower than that of JC-800-2. Furthermore, the average pore width for JC-900-4 is also lower compared to samples calcined at 800 and 900 °C for 2 h. This behavior may be associated with a starting sintering process, causing some pore size and surface area reduction. Sintering is a surface phenomenon taking place at high temperatures, which is dependent on the material characteristics, reducing the distance between grain centers, and indirectly reducing the surface area.^4,30,31^

**Table 3:** BET Surface Area and Porosity

sample	BET surface area (m^2^/g)	Langmuir surface area (m^2^/g)	adsorption average pore width (Å)
JC-0	1.35	1.89	10.75
JC-700-2	2.03	5.19	21.44
JC-700-4	1.48	2.36	7.07
JC-800-2	2.77	5.93	22.37
JC-800-4	2.02	4.42	14.73
JC-900-2	2.10	4.30	19.61
JC-900-4	2.65	5.36	14.22

In comparison to other calcined biomass samples, the calcined JCS exhibits a smaller surface area,^30,32^ particularly when compared to reported metal oxides,^33^ although it surpasses some CaO-based catalysts.^34^ The observed increase in surface area with the increase of calcination temperature has been reported for the case of solid catalysts derived from biomass waste.^35,36^ Furthermore, it has been established that the biodiesel yield shows a strong correlation with the BET surface area.

Figure 7a represents the BET adsorption–desorption isotherm of nitrogen gas at standard temperature and pressure (STP), for sample JC-700-2. The rest of the analyzed samples showed similar isotherms. This behavior reveals, according to the hysteresis loop, that there is probably a presence of a mesoporous catalyst surface. At low relative pressure (*p/p*_*o*_), the adsorption isotherm shows a progressive increase in the volume of N_2_ adsorbed, revealing a form of type IV isotherm (the slight decrease observed between relative pressures 0.5–0.8 could be attributed to percolation phenomena^37^). In the case of the nonactivated biomass sample, there was no regular nitrogen adsorption on the catalyst surface, indicating that the biomass precursor without thermal activation is not an adsorbent material, no matter if it is porous, justifying the thermal biomass treatment before its use as heterogeneous catalyst. Figure 7b shows the surface area plot, which relates 1/[*X·*(*p*_0_/*p*)*−*1] vs *p*/*p*_0_, where *X* is the adsorbed gas weight, resulting from the Multipoint BET method described by Pavan et al.^37^ The linear trend with a positive slope allowed us to obtain the surface area with enough accuracy. Similar behavior was observed in the rest of the samples.

**Figure 7:**
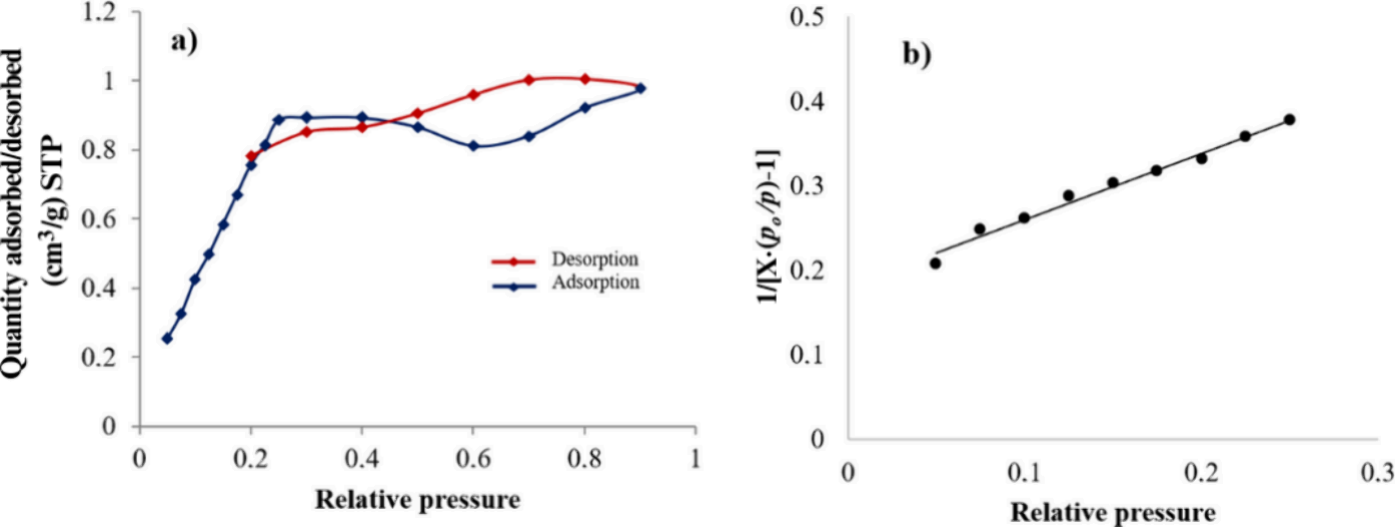
(a) Quantity of nitrogen gas adsorption/desorption on JC-700-2 surface vs relative pressure using the BET method. (b) BET surface area plot corresponding to JC-700–2.

The results of SEM are shown in Figure 8, corresponding to different calcination temperatures at 2 h. SEM analysis on the catalyst helps identify how changes in the calcination temperature will affect the surface composition and structure of the biomass. All sample images in this figure were taken at the same magnification. The morphology observed shows irregularities on the surface structure. Porosity changes may be associated with the release of CO_2_ and volatiles from the internal structure, also related to the transformation of carbonates on the surface into oxides. Calcination occurs reducing the volume of the particles, enhancing the formation of pores.^4^ As observed, each calcination temperature generates different surface morphologies. A quite different change in morphology is observed at 900 °C compared to those at 700 and 800 °C, resulting in small aggregates of fine particles. Particle structures of this nature typically exhibit a higher surface area, suggesting good catalytic activity of the developed catalyst. Similar behavior has been reported for calcination of different biomass types such as cocoa pod husk,^38^ ripe and unripe plantain peel,^39^ and kola nut pod husk.^40^

**Figure 8:**
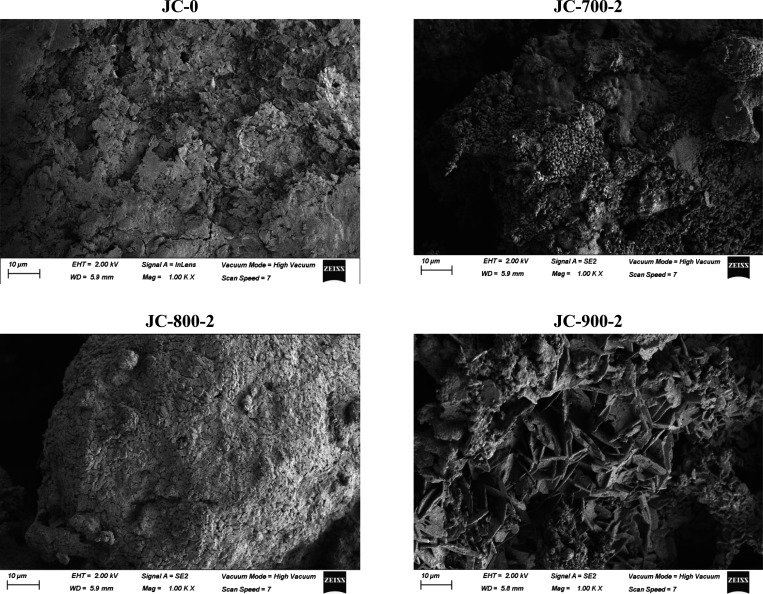
SEM images of JC samples calcined at different temperatures for 2 h.

The same surface morphology shown in Figure 8 for JC-900-2 was also observed for JC-900-4 and JC-800-4. This morphology may be associated with a more intense calcination process for the tested biomass. A higher surface could enhance the catalytic activity, but it is dependent not only on surface area but also on the composition (in this case mainly metal oxides) and number of active sites and the movement of the solid particles during the transesterification inside the reactor, enhancing or not the mass and heat transfer.

Regarding the EDX analysis, Table 4 displays the chemical elements identified on the surface of the precursor biomass and catalyst samples along with their elemental composition. The primary elements present in the uncalcined JC shell are C, O, and K. The application of calcination affects the concentration of elements compared to the initial biomass composition, as expected. The concentration of elements C and O is significantly reduced during calcination. The main elements present in the catalyst samples are K, Ca, Mg, and Si.

**Table 4:** Elemental Composition of the Samples Obtained with EDX

	**elemental composition (wt %)**						
**chemical elements**	**JC-0**	**JC-700-2**	**JC-700-4**	**JC-800-2**	**JC-800-4**	**JC-900-2**	**JC-900-4**
C	53.03	8.82	7.65	7.88	10.40	4.82	8.28
O	38.02	32.65	33.16	32.25	32.78	33.34	34.69
Na	0.34	1.82	2.10	2.25	2.94	2.31	2.45
Mg	0.97	5.16	5.89	4.63	3.30	6.46	3.80
Si	1.37	2.46	3.50	5.56	4.27	6.61	4.35
P	0.09	0.78	0.98	1.20	1.46	1.44	0.98
S	0.08	0.33	0.28	0.12	0.25	0.36	0.08
Cl	0.09	1.10	1.31	1.21	0.78	0.26	0.00
K	4.50	35.93	35.09	34.21	35.66	29.13	37.60
Ca	1.23	7.05	6.40	7.83	5.87	12.81	5.92
Fe	0.00	3.62	3.66	2.67	1.83	2.47	1.86

Increasing the calcination time from 2 to 4 h does not bring about substantial changes in the elemental composition, especially at 700 and 800 °C. Therefore, doubling the calcination time does not seem justified. However, at the maximum calcination temperature, the changes are more noticeable.

Figure 9 shows the increasing trend in the relative content of different elements (in percentage) at different calcination temperatures. The concentration of all elements (generally denoted as *i*) under any calcination condition exhibits an increasing trend compared with untreated biomass. Specifically, in the case of 2 h of calcination, the sample calcined at 900 °C displayed a lower relative content of the element K compared to samples activated at lower temperatures and a higher relative content of the elements Ca, Mg, and Si. This agrees with other reports about biomass calcination, where K shows the highest content but with significant amounts of Ca and Mg.^30^

**Figure 9:**
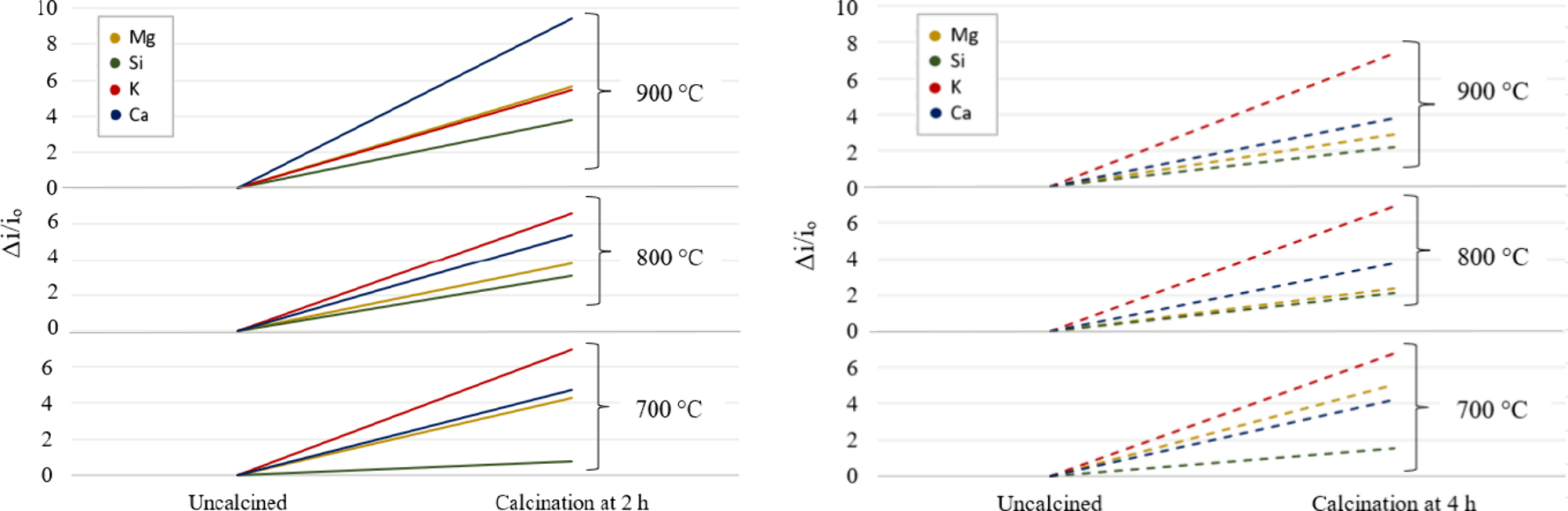
Influence of the calcination application on the relative elemental composition.

These results could suggest selecting more intense calcination conditions as optimal to increase the number of active metal sites on the catalyst surface. However, the increase in the relative content of elements on the catalyst surface does not necessarily imply better catalytic activity in the transesterification reaction as it depends on the form in which the elements are present. The main purpose of calcination of the sample is precisely the decarbonization of the biomass to favor the emergence of metal oxides that act as active sites.

The mineral phases present in the samples calcined at 700, 800, and 900 °C were identified from the XRD pattern shown in Figures 10, 11, and 12, respectively, by comparing with standard XRD powder patterns by National Bureau of Standards (NBS) and reported literature.^24,41–44^ The catalyst analysis reveals a poly mineral mixture of metal oxides such as K, Ca, Mg, and Si and metal carbonates such as K, Mg, and Ca, along with other mineral compounds.

**Figure 10:**
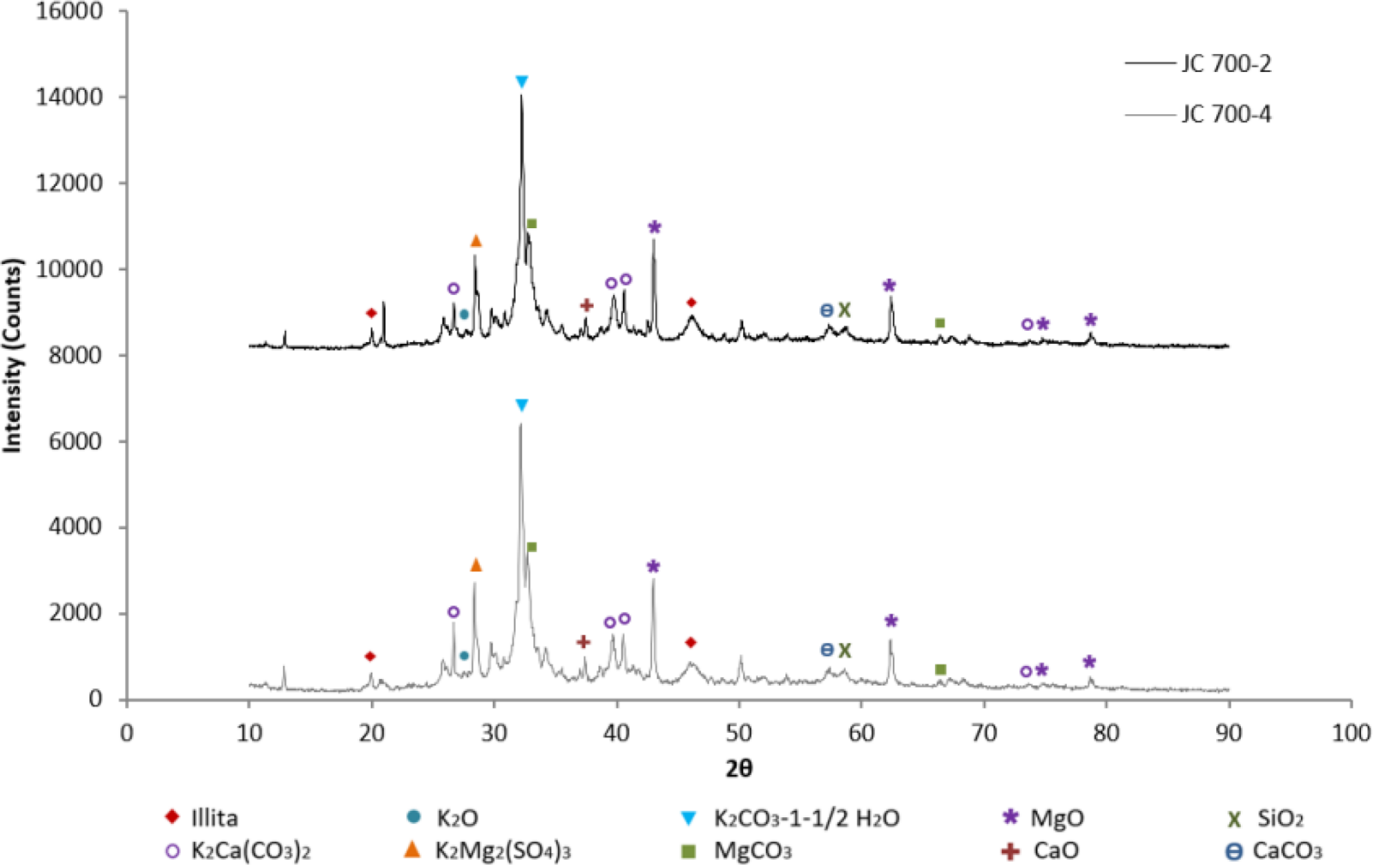
XRD results for catalyst at 700 °C

**Figure 11:**
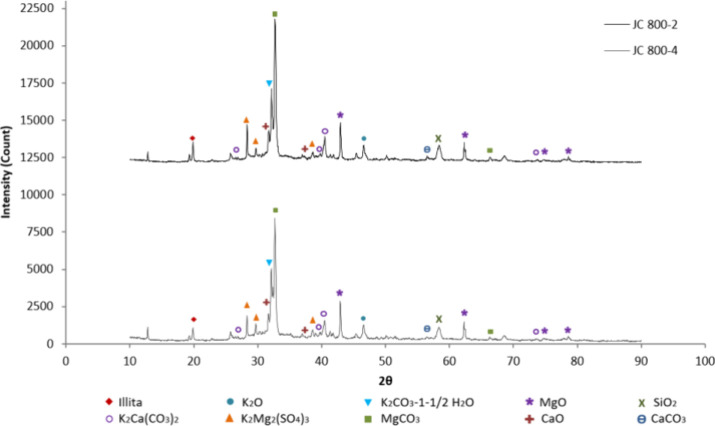
XRD results for the catalyst at 800 °C.

**Figure 12:**
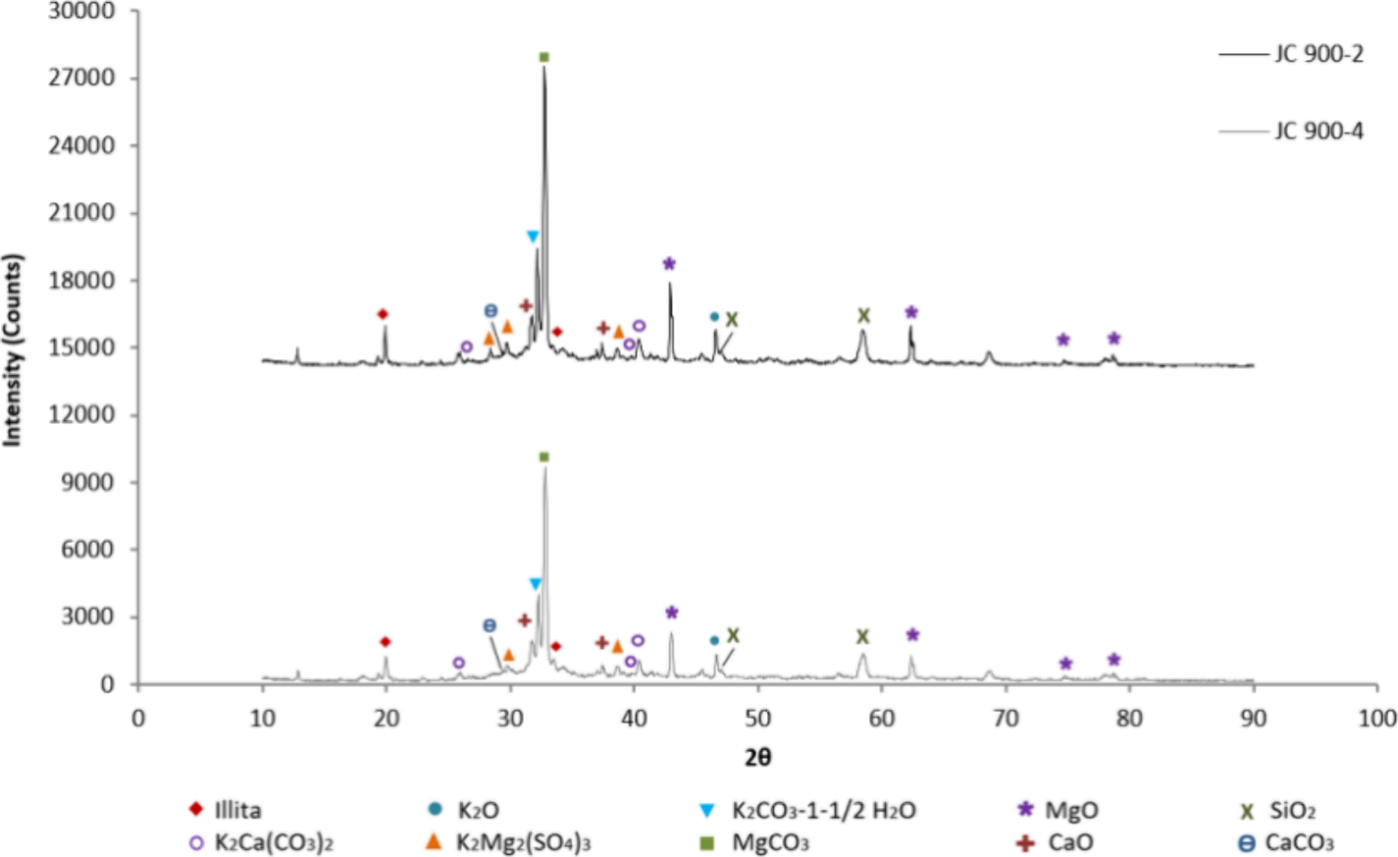
XRD results for the catalyst at 900 °C.

By comparison of the XRD patterns of each catalyst sample (Figures 10–12), it is evident that the peak intensities of the identified mineralogical phases vary according to the experimental conditions. For example, alterations in the activation conditions of the solid catalyst not only affect morphology, structure, surface area, and pore size, as previously discussed, but also modify the concentration of mineralogical phases. Specifically, the intensity of any peak in the diffractogram (peak height or area) depends on the concentration of that phase in the sample, making it possible to determine the mass fraction of the crystalline phase in the sample. Table 5 shows the mass fractions associated with each mineral compound. The mass fraction of mineral compound i (*x*_*i*_) is defined as

xi=AiRPi∑iAiRPi 6

where *A*_*i*_ is the area of the highest intensity peak of each mineral compound, and *RP*_*i*_ is its reflectance power.

**Table 5:** Quantitative Analysis from XRD^a^

			mass percentage (%) *x*_*i*_·**100**					
mineral phase (i)	molecular formula	**RP**_**i**_	JC-700-2	JC-700-4	JC-800-2	JC-800-4	JC-900-2	JC-900-4
illite	(K,H_3_O)(Al,Mg,Fe)_2_(Si,Al)_4_O_10_[(OH)_2_,(H_2_O)]	1.0	9.30	8.19	7.71	6.25	10.32	6.70
magnesium carbonate	MgCO_3_	1.87	10.00	11.82	43.80	38.49	43.80	47.72
calcium carbonate	CaCO_3_	3.38	1.62	2.01	0.54	0.37	0.67	0.65
potassium carbonate hydrate	K_2_CO_3_.1–1/2H_2_O	1.02	60.54	54.99	27.17	34.27	23.40	22.92
magnesium oxide	MgO	3.36	7.40	7.82	5.11	5.50	6.12	6.19
calcium oxide	CaO	4.83	0.69	1.05	2.39	2.12	3.36	3.42
potassium oxide	K_2_O	5.53	0.57	0.00	1.32	1.35	1.70	1.22
silicon oxide	SiO_2_	3.06	0.96	1.90	2.45	4.45	4.61	6.45
bütschliite	K_2_Ca(CO_3_)_2_	2.45	2.72	4.48	3.54	3.93	3.79	2.79
langbeinite	K_2_Mg_2_(SO_4_)_3_	2.19	6.21	7.74	5.97	3.28	2.23	1.92

a RP: Reflective power.

Although the compounds with the highest mass percentages are K_2_CO_3_.1–1/2H_2_O and MgCO_3_, as shown in Table 5, there is also a trend indicating that the mass fractions of metal oxides, such as CaO, K_2_O, and SiO_2_, increase, while the mass fractions of metal carbonates, particularly CaCO_3_ and K_2_CO_3_.1–1/2H_2_O, decrease with increasing calcination temperature. This behavior can influence the catalytic activity, making it essential to study the effect of calcination conditions on the concentrations of these carbonates and oxides. Figure 13 shows the influence of the calcination temperature on the mass percentage of these compounds in the sample.

**Figure 13:**
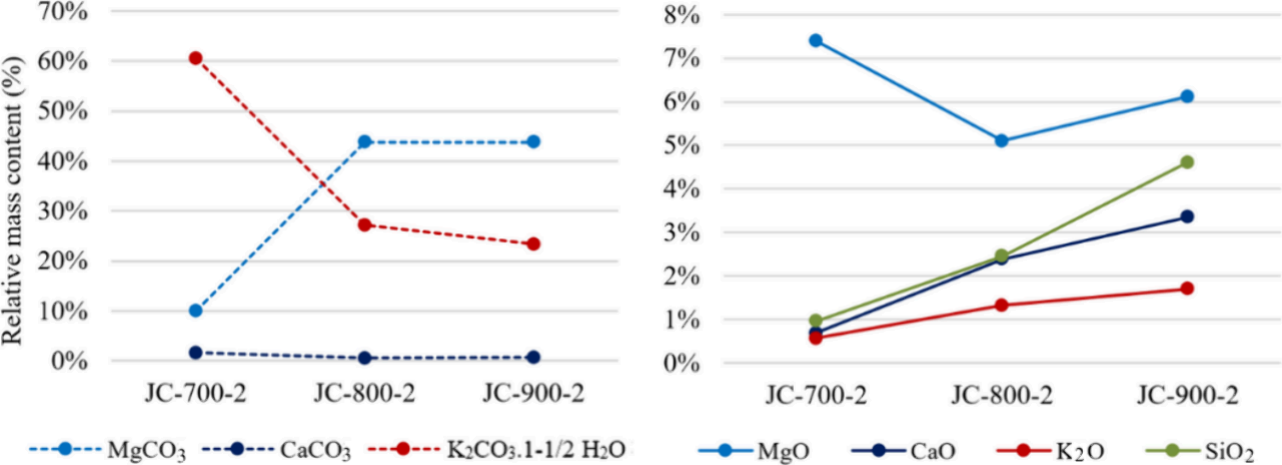
Influence of calcination temperature on the concentration of oxide and carbonate compounds

When the temperature is increased to 800 °C, there is a substantial decrease in K_2_CO_3_.1–1/2 H_2_O and CaCO_3_ by more than 50 and 60%, respectively, along with an increasing trend for K_2_O, CaO, and SiO_2_ to more than double, compared to the mass content at 700 °C. This indicates that the decarbonization process is favored. However, at the same time, the relative mass content of MgCO_3_ increases substantially with a 30% decrease in MgO, indicating the occurrence of MgO carbonation due to the reaction between MgO and CO_2_, which occurs rapidly during the catalyst’s exposure to air. In the second temperature interval (from 800 to 900 °C), the mass content of MgCO_3_ remains constant. The MgO increases, although to values lower than at 700 °C. The K_2_CO_3_.1–1/2 H_2_O continues to decrease, consistent with the results of EDX. The trend of the metal oxides is increasing.

As a result of the combined analysis of BET, SEM/EDX, and XRD techniques, it is possible to rule out the minimum calcination temperature (700 °C) and the higher calcination time value (4 h) as optimal calcination conditions. Increasing the calcination time 2-fold does not compensate, considering that it does not bring significant changes in the elemental composition of the catalyst, especially at 700 and 800 °C. Additionally, there is a decrease in the BET surface area and pore size due to the possible occurrence of sintering phenomena. On the other hand, at 700 °C, lower BET surface area values are obtained compared to higher temperatures, and the concentration of metal oxides is minimal. However, to select the optimal calcination conditions, it is important to evaluate the effectiveness of the catalyst samples in biodiesel production at the laboratory scale.

### 4.3 Biodiesel Production

#### 4.3.1 Esterification Reaction

The acid value of JCO esterified with *p*-toluenesulfonic acid is 1.4 mgKOH/g, and with H_2_SO_4_, it is 1.3 mgKOH/g, resulting in esterification conversions of 81.96 and 83.25%, respectively. Considering that the effectiveness in reducing the acid value is very similar for both acids, with only a difference of 0.1 mgKOH/g, and that using *p*-toluenesulfonic acid increases the esterification reaction time to 4 h compared to H_2_SO_4_, which completes in 1 hour, H_2_SO_4_ is selected as the acid catalyst. Subsequently, JCO esterified with H_2_SO_4_ (section 3.3.1) proceeds to the second reaction stage via heterogeneous catalytic transesterification.

#### 4.3.2 Transesterification Reaction

Tables 6 and 7 show the ester content values and the fatty acid profile of the FAME samples resulting from the transesterification of SFO and JCO, respectively (FAME-SFO and FAME-JCO). The conversion and yield of FAME for each experimental condition are listed in Figure 14. The values obtained for ester content, conversion, and yield of FAME when using JCO are more promising than those for SFO. As shown in Table 2, JCO contains a lower proportion of unsaturated fatty acids than SFO. The higher degree of unsaturation in the molecules could hinder their access to the catalyst pores due to their stereochemistry, affecting the catalytic performance. Additionally, the FAME yields correspond to the pure isolated fuel, and in this context, other factors such as viscosity and water solubility may also contribute to the observed differences in Tables 6 and 7.

**Table 6:** Ester Content and Fatty Acid Profile of FAME-SFO^a^

			**fatty acid profile (wt %)**								
**catalyst sample**	**catalyst amount (wt %)**	**C (wt.%)**	**C**16:0	**C**18:0	**C**18:1	**C**18:2	**C**20:0	**C**22:0	**C**24:0	**SFA**	**UFA**
**JC-700-2**	1	85.88	5.13	3.18	82.26	7.39	0.32	1.05	0.37	8.63	77.25
**JC-800-2**	1	83.19	4.96	3.18	82.75	7.32	0.35	1.06	0.39	8.26	74.93
**JC-900-2**	1	68.52	4.94	3.06	82.67	7.12	0.48	0.93	0.80	7.00	61.52
**JC-700-2**	3	88.72	5.26	3.24	82.13	7.32	0.31	1.06	0.38	9.10	79.63
**JC-800-2**	3	90.25	5.02	3.17	83.02	7.33	0.00	1.07	0.39	8.71	81.55
**JC-900-2**	3	89.71	5.09	3.23	82.47	7.40	0.32	1.09	0.40	9.08	80.63
**JC-700-2**	5	91.31	5.04	3.21	82.56	7.36	0.32	1.11	0.40	9.21	82.10
**JC-800-2**	5	91.13	4.97	3.23	83.21	7.06	0.00	1.13	0.40	8.87	82.26
**JC-900-2**	5	90.41	5.04	3.22	82.96	7.32	0.00	1.07	0.40	8.79	81.62

a C: Ester content determined by standard EN 14103; SFA: saturated; UFA: unsaturated.

**Table 7:** Ester Content and Fatty Acid Profile of FAME-JCO^a^

			**fatty acid profile (wt %)**										
**catalyst sample**	**catalyst amount (wt %)**	**C (wt %)**	**C**16:0	**C**16:1	**C**18:0	**C**18:1	**C**18:2	**C**18:3	**C**20:0	**C**22:0	**C**24:0	**SFA**	**UFA**
**JC-700-2**	1	83.40	14.30	0.86	6.90	41.00	36.45	0.24	0.25	0.00	0.00	21.45	78.55
**JC-800-2**	1	92.10	14.35	0.86	6.67	42.21	35.91	0.00	0.00	0.00	0.00	21.02	78.98
**JC-900-2**	1	89.06	14.41	0.86	6.77	41.67	35.85	0.23	0.21	0.00	0.00	21.39	78.61
**JC-700-2**	3	92.09	14.42	0.87	6.74	40.70	36.80	0.23	0.24	0.00	0.00	21.40	78.60
**JC-800-2**	3	95.66	13.66	0.81	6.75	42.87	34.19	0.22	0.22	0.31	0.86	21.79	78.09
**JC-900-2**	3	90.45	14.40	0.87	6.74	40.76	36.75	0.23	0.11	0.14	0.00	21.40	78.60
**JC-700-2**	5	90.60	14.46	0.87	6.72	40.77	36.85	0.23	0.11	0.00	0.00	21.28	78.72
**JC-800-2**	5	89.96	14.37	0.86	6.78	40.80	36.52	0.22	0.23	0.11	0.11	21.59	78.40
**JC-900-2**	5	92.62	14.58	0.88	6.67	40.51	36.66	0.22	0.26	0.11	0.12	21.73	78.27

a C: Ester content determined by standard EN 14103; SFA: saturated; UFA: unsaturated

**Figure 14:**
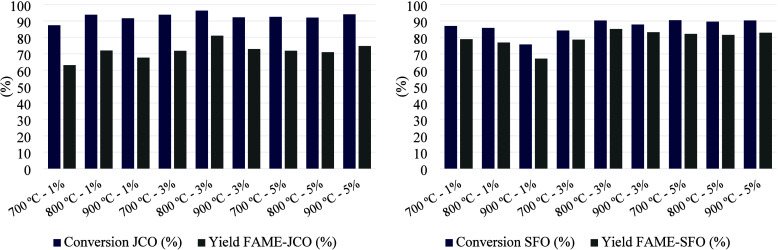
Conversion and FAME yield.

Table 6 shows that the minimum ester content values correspond to the lowest catalyst loading (1%). An increase in catalyst loading in the reaction favors the ester content, although there are no substantial changes between 3 and 5% loading for FAME-SFO. However, for the FAME-JCO samples (Table 7), at the minimum catalyst loading, conversion values between 83 and 92% were obtained, slightly higher than those for FAME-SFO. The maximum ester content value was 95.66% and corresponds to the FAME-JCO sample obtained with 3% of JC-800-2 catalyst. According to the EN 14103 standard, the minimum ester content in pure biodiesel must be above 96.5%.^4^ Nonetheless, this is a promising result as a starting point in the development of the catalyst.

As shown in Figure 14, most of the conversion values for JCO are between 90 and 94%, except for the low level of the experimental design (700 °C and 1%), for which the minimum conversion value was obtained (87.41%), and for the medium level of the design (800 °C and 3%), for which the maximum conversion (96.31%) was achieved. However, for SFO, the conversion does not exceed 90% in any case.

In theory, a higher catalyst loading favors conversion of the transesterification reaction. However, this study demonstrates that this is not necessarily the case. Increasing the catalyst loading in the reaction from 1 to 3% influences positively the conversion achieved in the transesterification reaction, but when the loading is 5%, the conversion values decrease. This same behavior is observed in the FAME yield values. This can be explained because at the same stirring speed, when the amount of catalyst in the reaction increases, small solid particles combine with each other, forming agglomerates that result in larger particles. An increase in particle size can lead to a decrease in surface area and, consequently, to a direct reduction in active sites in contact with the reaction media.^10,45–47^ Additionally, when there is more solid catalyst in the reaction, it is likely that the stirring speed needs to be higher to keep all of the catalyst in suspension and favor the reaction.

On the other hand, analyzing the effect of calcination temperature, the highest conversion and FAME yield values were obtained at 800 °C. This result corresponds to the variations in surface area observed in Table 3, demonstrating the direct relationship between surface area and catalytic activity. It has been reported that solid catalysts with comparable surface areas have similar or even lower FAME yields compared to the yield obtained in the current study, even using significantly higher catalyst loadings in some cases.^33,34^ Table 8 summarizes several reports on the use of heterogeneous solid catalysts from different precursors for biodiesel production.

**Table 8:** Solid Catalyst Used for Biodiesel Production

catalyst source	conditions calcination	*S*_BET_ (m^2^/g)	methanol/oil molar ratio	catalyst loading (wt.%)	temperature (°C)	time (min)	yield or conversion (%)
J. curcas shell (this study)	800 °C, 2 h	2.77	15:1	3	60	120	96.31^C^ 81.4^Y^
Sesamum indicum^43^	550 °C, 2 h	3.66	12:1	7	65	40	98.90^Y^
sugar beet agro-industrial waste^48^	800 °C, 2 h	27.9	4.5:1	1	75	60	93.00^C^
crab shell^49^	900 °C, 2 h	1.93	6:1	3	60	240	83.10^Y^
eggshell^49^	900 °C, 2 h	1.60	9:1	3	60	240	97.75^Y^
eggshell^50^	800 °C, 4 h	5.70	9:1	3	65	240	90–95^Y^
walnut shell^51^	800 °C, 2 h	8.80	12:1	5	60	120	98.00^Y^
oyster shell^52^	850 °C, 3 h	15.6	6:1	2	60	240	89.20^C^
rice husk-derived sodium silicate^45^	300 °C, 1 h	1.14	12:1	2.5	65	30	97.00^Y^
carbonaceous residue^53^	500–800 °C, 4 h	<5.00	30:1	10	80	720	75.00^C^
CaO^54^	1000 °C	32.00	13:1	3	60	100	94.00^Y^
CaO^55^	300–900 °C, 2 h		6:1	1	60	120	98.00^Y^

The results of this study are promising, even exceeding some reported values, and considering that there is still potential to increase ester content, conversion, and FAME yield through optimization of the process operating conditions. Compared to most reports,^10,56^ the conversion using the JCS catalyst involves a lower amount of catalyst and reaction time, with a similar reaction temperature.

Summarizing, the best conversion and yield values correspond to the experimental conditions that, according to the characterization techniques described (BET, SEM/EDX, and XRD), promote catalytic activity. TGA was the starting point that allowed for defining the temperatures used for the calcination of the precursor biomass (700, 800, and 900 °C), enhancing the formation of oxides that favor the appearance of active sites in the catalyst. However, it is the combined analysis of all of the applied characterization techniques that allows for selecting the optimal conditions. The JC-800-2 catalyst sample not only reported the best conversion and yield values but also presented the highest surface area and pore width, according to BET and SEM results, confirming the above-mentioned direct relationship between morphology and catalytic activity. Moreover, the XRD results complemented the EDX analysis, as it was demonstrated that an increase in the relative content of chemical elements on the surface of the catalyst does not necessarily imply better catalytic activity in the transesterification reaction, as it depends on the form in which the elements are present. The XRD analysis indicates that the content of metal oxides increases as the calcination temperature rises. However, at the highest calcination temperature, a decrease in FAME conversion becomes evident, probably due to sintering, which hinders the contact between the TGs and the active sites on the catalyst surface. This is reflected in the catalytic process quality and reaction performance. Therefore, the JC-800-2 sample with 3% w/w catalyst exhibited the maximum conversion to FAME under the established reaction conditions.

The use of heterogeneous catalysts in the synthesis of biodiesel offers a key advantage in terms of recoverability and reuse, thus contributing to the reduction of costs in biodiesel production. However, in the process of catalyst recovery and reuse, there is a potential challenge: the agglomeration of ester, glycerol, and FFA molecules on the surface of the catalyst.^40,51,57^ This agglomeration can lead to blocking of active sites on the catalyst along with the risk of leaching. Consequently, the conversion efficiency of biodiesel is compromised, due to reduced stability and catalytic activity.^48,44^ These factors limit the reuse potential of the solid catalyst. In this case, the recovered catalyst was reused in a second reaction cycle, showing a significant decrease in ester content to only 78.6%, indicating deactivation. In most processes, the loss of catalytic activity of solid catalysts is unavoidable, so catalyst reactivation between recycles appears necessary.^10^ Reactivation of the catalyst was by calcination at 800 °C for 2 h. Reactivation restored catalytic activity with the ester content increasing up to 90.7%. However, the catalytic performance progressively declined in subsequent cycles. After the third recycling, catalytic activity decreased again, limiting conversion to 72%, and in the fourth cycle, the ester content, the oil conversion, and the yield decreased sharply to below 50%. These results are consistent with other studies where reactivation restored initial catalytic activity, but conversion generally decreased in subsequent cycles.^10,48,51^

Future work can focus on the reactivation stage, exploring alternatives to high-temperature calcination or using less aggressive conditions to avoid sintering, which collapses the catalyst’s porous structure, limiting its effectiveness. This could help enhance the long-term reusability of solid catalysts in biodiesel production.

## 5 Conclusions

The use of calcined JCS as a heterogeneous catalyst for biodiesel production was studied in this work. The TGA allowed defining the temperatures used for calcination of the precursor biomass, enhancing the formation of oxides that favor the appearance of active sites in the catalyst and, therefore, better catalytic activity. The surface area and pore width increased with increasing calcination temperature from 700 to 800 °C. However, at 900 °C, these values decreased, which may be due to the occurrence of inceptive sintering. The surface structure of the catalyst shows an irregular morphology with changes in porosity, associated with the release of CO_2_, volatiles, and decarbonization process. A different surface structure is observed under more intense calcination conditions (JC-800-4, JC-900-2, and JC-900-4), compared to small fine particle aggregates at JC-700-2, JC-700-4, and JC-800-2. According to the EDX analysis, the main elements present on the catalyst surface are K, O, Ca, Mg, and Si. The XRD patterns show that as the calcination temperature increases, the presence of MgO, CaO, SiO_2_, and K_2_O also increases. Calcination for 4 h does not produce significant changes in the chemical composition of the catalyst. Esterifying JCO with H_2_SO_4_ ensures an acid value of 1.3 mgKOH/g, like that obtained with *p*-toluenesulfonic acid (1.4 mgKOH/g), but less time is needed to complete the reaction. The values obtained for ester content, conversion, and FAME yield are more promising when JCO is used in a two-step reaction compared with SFO in a single-stage reaction. A 3% catalyst obtained by calcination at 800 °C for 2 h resulted in optimal conditions that ensure better catalytic activity during transesterification.

## Data Availability

The data sets generated during the current study are available at 10.17632/672n24fx8n.1.
